# Entropy as a Lens: Exploring Visual Behavior Patterns in Architects

**DOI:** 10.3390/jemr18050043

**Published:** 2025-09-16

**Authors:** Renate Delucchi Danhier, Barbara Mertins, Holger Mertins, Gerold Schneider

**Affiliations:** 1 Psycholinguistics Laboratories, TU Dortmund University, 44227 Dortmund, Germany; barbara.mertins@tu-dortmund.de; 2 KRESINGS Architektur Münster GmbH, 48155 Münster, Germany; holger.mertins@web.de; 3 Department of Computational Linguistics, University of Zurich, 8006 Zürich, Switzerland; gerold.schneider@es.uzh.ch

**Keywords:** eye-tracking, experts, architecture, entropy, visual

## Abstract

This study examines how architectural expertise shapes visual perception, extending the “Seeing for Speaking” hypothesis into a non-linguistic domain. Specifically, it investigates whether architectural training influences unconscious visual processing of architectural content. Using eye-tracking, 48 architects and 48 laypeople freely viewed 15 still images of built, mixed, and natural environments. Visual behavior was analyzed using Shannon’s entropy scores based on dwell times within 16 × 16 grids during the first six seconds of viewing. Results revealed distinct visual attention patterns between groups. Architects showed lower entropy, indicating more focused and systematic gaze behavior, and their attention was consistently drawn to built structures. In contrast, laypeople exhibited more variable and less organized scanning patterns, with greater individual differences. Moreover, architects demonstrated higher intra-group similarity in their gaze behavior, suggesting a shared attentional schema shaped by professional training. These findings highlight that domain-specific expertise deeply influences perceptual processing, resulting in systematic and efficient attention allocation. Entropy-based metrics proved effective in capturing these differences, offering a robust tool for quantifying expert vs. non-expert visual strategies in architectural cognition. The visual patterns exhibited by architects are interpreted to reflect a “Grammar of Space”, i.e., a structured way of visually parsing spatial elements.

## 1. Introduction

Understanding how individuals visually engage with architectural environments is central to both cognitive science and the design of user-centered spaces. As of 2025, more than 58% of the global population (approximately 4.8 billion people) live in urban areas, a figure that has steadily increased since the urban population surpassed the rural population globally in 2007. This growing urbanization means that modern individuals spend most of their lives surrounded by built environments that shape how they perceive, navigate, and interact with space. Despite the omnipresence of architectural environments in daily life, relatively little is known about how architectural expertise modulates visual attention during real-time scene perception. A particularly underexplored question is how architects and laypeople differ in their visual processing of architectural scenes.

Most empirical studies in architecture rely on evaluative methods such as questionnaires or preference ratings, e.g., asking participants to compare wooden versus concrete façades. While such methods provide insight into subjective judgments, they fail to capture the dynamic and exploratory nature of visual engagement in complex environments. Eye-tracking research offers a promising alternative by allowing a direct measurement of visual attention in real time. However, many eye-tracking studies rely on predefined Areas of Interest (AOIs), which impose artificial and often unvalidated structures onto gaze data. This is especially problematic when comparing groups with differing expertise: AOIs that seem meaningful to researchers may not align with what architects or laypeople find perceptually salient.

To overcome this limitation, we introduce an entropy-based analytic framework that allows for an AOI-independent analysis of gaze behavior. Entropy quantifies how visual attention is distributed across a scene, offering an objective metric for attentional dispersion and focus. This method captures both exploratory and selective viewing patterns without relying on pre-categorized visual elements. It thus allows for a more ecologically valid comparison between expert and non-expert observers. By using entropy, we aim to capture the fluid and individualized nature of visual exploration in complex visual scenes.

Our study investigates how architectural expertise shapes visual attention and spatial cognition. We conducted a controlled eye-tracking experiment with 48 trained architects and 48 laypeople, who viewed a diverse set of high-resolution photographic scenes. These included images of urban architecture, buildings integrated into natural landscapes, and control scenes containing no architectural elements. To maintain engagement and standardize task demands without biasing visual attention, participants answered unrelated filler questions between trials. This design allows us to examine spontaneous gaze behavior as modulated by domain-specific knowledge.

The paper is structured as follows: Section 2 reviews relevant theoretical and empirical work on expertise and visual attention. Section 3 outlines our methodological approach and data analysis. Section 4 presents the results of the entropy analysis, and Section 5 discusses the implications of our findings for spatial cognition, architectural design, and eye-tracking methodology.

## 2. Literature Review

### 2.1. Expertise Acquired Through Architectural Education and Training

Architectural education fosters a specialized understanding of the multifaceted nature of space and its underpinnings. Through rigorous and systematic training in spatial thinking, design principles, and the interplay between built environments and human behavior, architects develop an acute awareness of the organization, flow, and aesthetics of spaces. Architectural education emphasizes the dynamic interactions between people and their environments, framing space as an experiential domain rather than a static container. Circulation paths, transitions, and spatial relationships are deliberately designed to evoke specific emotions and reactions.

Architects are trained to analyze space through multiple lenses (functional, experiential, and symbolic), enabling them to discern layers of meaning and intentionality embedded in architectural design that transcend mere functionality [1,2]. In an architectural context, the fictional sense refers to how the space fulfils its intended purpose, the experiential sense to the emotional and psychological impact on the users of the space, and the symbolic sense to the cultural or metaphorical significance embedded in architectural design. Architects possess specialized knowledge of design elements, proportions, and historical contexts of buildings and public spaces, allowing them to notice details such as facade materials, the placement of street furniture, or pedestrian flow patterns that untrained observers might overlook. Their perspective extends beyond individual spaces to encompass the relationships between buildings, their interiors, and their broader environmental context. Interior spaces are designed in harmony with exterior views and natural light, while the interaction between buildings and landscapes reflects cultural and historical contexts. Architectural training emphasizes the conceptualization of space as dynamic, interactive, and user-centered. Architects design circulation paths to guide movement and craft transitions between spaces to evoke emotions or reactions, creating a cohesive spatial experience. This perspective underscores the importance of the interplay between users and the designed environment.

Previous research has highlighted the broader role of architectural design in shaping human experiences, such as how school building design influences learning [3]. The specific impact of architectural education on the perception of everyday environments, such as streets, has received little attention in academic research. We argue that architectural expertise cultivates heightened sensitivity to spatial relationships, design cues, and the interplay between built forms and their surroundings (built or natural). This unique lens, cultivated through training, allows architects to interpret and engage with the built world in ways that differ significantly from those without such training.

### 2.2. Some Previous Studies on Expertise

Cognitive expertise in spatial reasoning has been linked to neurobiological changes. For example, a neuro-imaging study [4] showed that a comparison of MRIs of the brains of 16 right-handed taxi drivers to those of a control group consisting of non-drivers exhibited an altered structure of the posterior hippocampus in the taxi drivers. Given the hippocampus’s role in spatial representation, this structural difference was attributed to navigational demands.

There have also been studies on the expertise of architects. For example, the opinions of architects have been found to be different from those of laypeople: Benz & Rambow [5] compared the oral evaluation of 65 architects and 75 laypeople regarding the use of exposed concrete, using interviews conducted in front of two buildings with examples of exposed concrete architecture. Results show that architects value the material and can recognize it even if it is painted, while laypeople value the material less and have trouble recognizing it, especially when it is painted.

The eye-tracking method has been used to study the visual attention of architects. A previous eye-tracking study using comparable stimuli [6] found significant differences in gaze patterns between architects and laypeople: 19 architects and a control group of 45 laypeople freely viewed five naturalistic pictures of the outdoors and six rendered images depicting scenes inside buildings before answering questions about the images. The analysis of the eye-tracking data was based on dwell times on areas of interest (AOI) in the six seconds of free looking. Group differences were statistically evaluated using *t*-tests on dwell times in predefined AOIs. In the scenes inside buildings, architects had significantly lower dwell times (ms) in the AOIs surrounding human figures compared to the group of laypeople. In the case of the outside stimuli (corresponding to the built environment category in this study), architects had significantly higher dwell times on the AOI defined over the cubature of buildings compared to the controls. Both results corresponded to medium effect sizes. They were interpreted to suggest that architects spent significantly more time fixating on architectural features, whereas laypeople focused more on the human occupants of interior scenes and explored exterior scenes primarily at pedestrian eye level. Another eye-tracking study [7] presented images of built environments to 24 architects and 24 laypeople. During the first 2 s of free viewing, architects exhibited significantly longer dwell times on elevated façade areas, building volumes and columns compared to laypeople. In contrast, laypeople showed greater interest in written elements such as advertisements and signage. These findings were also interpreted to indicate that laypeople tend to maintain a pedestrian-level perspective, while architects attend more to architectural features and geometric-visual effects, such as the perception of contours, depth cues, or patterns.

### 2.3. Entropy in Visual Attention

The theoretical framework for the analysis is grounded in Shannon’s information theory. In information theory, entropy serves as a measure of unpredictability or complexity in a system [8,9]. In this context, entropy quantifies the probability distribution of gaze positions, providing a proxy for attentional variability and structure: low entropy reflects highly predictable distributions with unequal probabilities, whereas high entropy corresponds to scenarios where all possibilities are equally probable, i.e., higher entropy values signify greater unpredictability, while lower values indicate structured and predictable patterns. Originally developed to optimize data compression in telecommunications, Shannon’s entropy has since become a versatile tool in fields such as linguistics, psychology, and neurolinguistics. When applied to eye-tracking data, higher Shannon entropy values reflect greater complexity and unpredictability in visual behavior. For instance, a high-entropy eye movement pattern in a classroom setting suggests that a teacher distributes their gaze equally among students, making it equally likely for any student to be looked at next [10]. In visual expertise research, experts often exhibit more complex scanpaths, characterized by higher entropy coefficients. This indicates frequent revisits to multiple areas of interest (AOIs), reflecting a more distributed visual attention strategy. Entropy can also be calculated for specific gaze behaviors: higher transition entropy signifies more frequent and random shifts between AOIs, while higher stationary entropy reflects more equal distribution of attention across AOIs. For example, Krejtz et al. [11] used eye-tracking to examine entropy in gaze behavior while laypeople viewed artworks under mood induction. A sample of 43 students viewed three works of art for 30 s each, after a positive or neutral mood was induced through sentences and music. The artworks were divided into two or three large Areas of Interest (AOIs). Eye movements were modeled using first-order Markov chains, demonstrating that a participant’s next fixation depended only on their current fixation. Two types of entropy were calculated: transition entropy, which measured the predictability of gaze transitions between AOIs (lower transition entropy indicated more focused viewing), and stationary entropy, which measured the evenness of gaze distribution across AOIs (higher stationary entropy indicated more evenly distributed attention). The study found that individuals with high curiosity exhibited lower transition entropy, suggesting more focused viewing patterns, while stationary entropy did not differ significantly between curiosity groups. Additionally, participants who rated the artwork more favorably tended to have lower transition entropy and higher stationary entropy, indicating deliberate and focused viewing paired with broader attention distribution. The authors concluded that entropy, particularly transition entropy, is a valuable metric for analyzing eye movement patterns and understanding visual attention.

The eye-tracking method has been used to study the visual attention of experts, quantifying the complexity of visual behavior using Shannon’s entropy of information. Kosel et al. [10] investigated scanpath patterns of 44 teachers (35 novice teachers and nine expert teachers) during an assessment task. Participants watched a video of a classroom and were subsequently asked to assess five different students. The researchers found that, consistent with prior research, scanpaths were more similar within an individual than between individuals, indicating idiosyncratic visual behavior. Further, experts’ scanpaths were more complex, involving more frequent revisits of all students, which was quantified using Shannon’s entropy score. Importantly, experts’ scanpaths were more similar to those of other experts within the group, and experts’ visual behavior was statistically linked to higher judgment accuracy.

Recent studies on the application of entropy measures on eye-tracking data [12] have shown that while high or low entropy values can characterize fixations, the interpretation of these metrics is highly dataset-dependent: Melnyk et al. [12] evaluated six entropy metrics (fuzzy, increment, sample, gridded distribution, phase, and spectral entropies) applied to fixation eye movement trajectories. In one dataset, high gridded distribution entropy and high fuzzy entropy correlated with high-frequency noise, potentially indicative of ocular microtremor. Conversely, in another dataset, low sample entropy was linked to fixations with significant linear drift. These findings underscore the importance of contextualizing entropy metrics, as their interpretation depends heavily on stimulus characteristics and task demands.

### 2.4. Seeing-for-Speaking

The overarching theoretical framework is grounded in the principle of linguistic relativity, also known as the Sapir–Whorf Hypothesis, which posits that language helps shape thought and cognition. Empirical and experimental evidence for linguistic relativity has been documented across various cognitive domains, including color cognition [13,14], motion cognition [15,16], gender cognition [17,18], numeral cognition [19,20], and spatial cognition [21,22,23].

Within the framework of linguistic relativity, the idea that grammatical features of a language influence the mental processes involved in conceptualization is known as the Thinking-for-Speaking Hypothesis [15]. Numerous studies [24,25] have demonstrated that conceptualization is language-specific rather than universal, with thinking processes shaped by the structure of the language being spoken.

The Seeing-for-Speaking Hypothesis [25,26,27,28] and the Seeing-for-Saying Hypothesis [29,30] extend the Thinking-for-Speaking framework by proposing that core grammatical features shape not only conceptualization but also visual attention and, consequently, perception. These findings are largely derived from eye-tracking studies, which effectively capture patterns of attention and conceptualization. Additionally, experimental studies suggest that the effects of grammar persist even in non-verbal tasks, such as memory tasks [28,31].

## 3. Materials and Methods

To investigate patterns of visual attention, we employed eye-tracking, a method well-suited to this purpose due to the largely automatic and involuntary nature of eye movements. Our study aimed to compare the gaze behavior of architects and non-architects (laypeople) during free viewing of scenes containing architectural content but without an explicit architectural task. Participants were asked to observe various images depicting architecture in different contexts and then respond to simple questions that were not relevant to the study’s core analysis.

The study was approved by the Ethics Committee of TU Dortmund University (GEKTUDO_2025-09) and conducted in accordance with the ethical standards outlined in the 2013 Declaration of Helsinki.

### 3.1. Stimuli

The stimuli were selected to elicit naturalistic scanning behavior in ecologically valid architectural contexts, rather than the highly constrained gaze patterns often found in laboratory settings. Unlike prior research that used rendered or artificially simplified visuals, we employed full-color photographs of real-world environments to enhance ecological validity and more accurately reflect the influence of architectural expertise on visual engagement.

We selected entropy modeling as our primary analytic tool, as it permits analysis of gaze distributions without reliance on predefined Areas of Interest (AOIs). This approach is especially well-suited for complex, naturalistic stimuli, as it accommodates visual variability and captures global patterns of exploration. Our goal was not to determine where viewers look under tightly controlled conditions, but rather to investigate how gaze behavior adapts to semantically distinct environments. This objective is best served by using non-standardized, but systematically categorized, stimuli that preserve the richness of real-world scenes.

During the initial pre-selection phase, we aimed to ensure some degree of visual consistency within each semantic category, particularly with respect to viewpoint and the prominence of architectural features. This reflects a deliberate trade-off favoring ecological validity over psychophysical standardization. As such, we did not control for psychophysical variables like brightness, contrast, visual complexity, number of focal elements, perspective, depth, or pixel-based image entropy. While high-entropy images contain a wide range of pixel values and appear more complex or textured, and low-entropy images appear more uniform, we did not formally calculate image entropy. Instead, candidate photographs were visually screened for comparable resolution, color quality, and dimensions.

However, while entropy quantifies complexity, it does not account for the semantic content of the image, i.e., the specific subject matter. For instance, a city skyline and a park may exhibit similar pixel-based entropy but differ significantly in their semantic interpretation. To verify the semantic classification of the stimuli, approximately 40 students completed a categorization task in which they assigned candidate images to one of three categories: built, natural, or mixed environments. A panel of five independent reviewers then evaluated these classifications and flagged inconsistencies. Only images that were consistently categorized were retained. This validation process confirmed the conceptual clarity of the categories while preserving the natural variability of real-world scenes.

**Figure 1:**
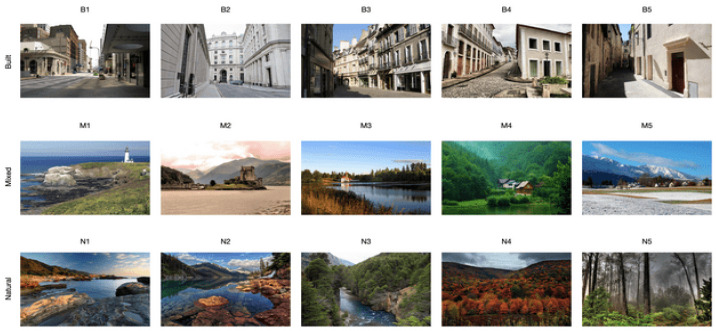
All 15 stimuli used in the study, grouped by category (Built, Mixed, Natural) based on the density of built elements.

The final stimulus set comprised 15 photographs, divided equally into three semantic categories: built urban environments (Built stimuli), natural landscapes (Natural stimuli), and scenes that combined architectural and natural elements (Mixed stimuli), with five images per category (Figure 1). This classification aimed to reflect ecologically valid contrasts while maintaining visual and thematic consistency within each category. Stimuli, raw data, and results are available as part of the Supplementary Materials.

### 3.2. Procedure

**Figure 2:**
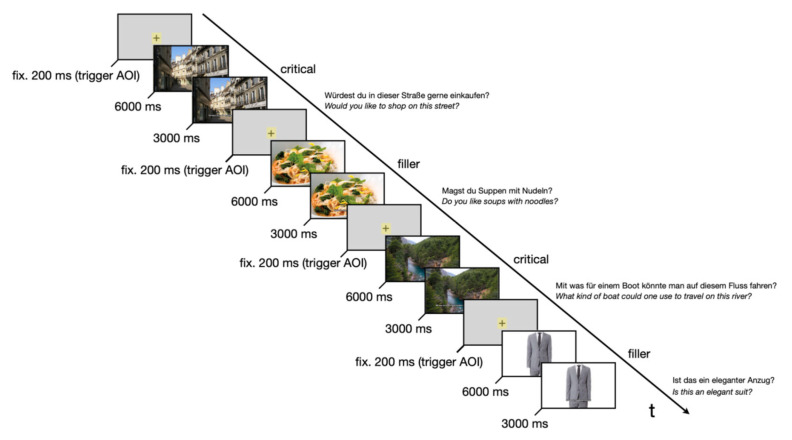
Overview of the experimental design.

Stimuli were presented as still images in randomized order on a monitor. Each image was shown for six seconds, followed by an overlaid caption at the bottom of the screen that posed a question. This caption appeared for three seconds. Participants were instructed to freely observe the image and then respond orally to the question. These questions, such as “Would you like to live or vacation here?” or “Does this seem like an expensive neighborhood?”, were unrelated to the study’s research goals. They functioned as a pseudo-task to keep participants engaged and ensure a consistent viewing pattern across trials. Responses were not recorded or analyzed. To further mask the critical stimuli and maintain task variability, we included 30 filler images depicting non-architectural content (e.g., clothing, food, sports). These followed the same structure: six seconds of free viewing followed by a pseudo-question. The experimental design is shown in Figure 2. All instructions and materials were presented in German, the native language of all participants. Materials have been translated into English for the reader’s convenience in this paper.

Stimuli were displayed on a 22-inch LCD monitor (1680 × 1050 resolution) connected to a standard PC. Eye movements were recorded using an SMI RED500 eye tracker in a controlled laboratory environment. Eye-movement data were exported using SMI’s BeGaze software, and all subsequent analyses were performed in R [32].

The first 250 ms after image onset were excluded from analysis, as participants were required to fixate on a central cross prior to stimulus presentation. This procedure ensured standardized starting conditions but introduced a pronounced center bias in the initial fixations. Therefore, the analyzed time window was 0.25 to 6.00 s. This time window also aligns with related studies [6,7], improving comparability. After six seconds, participants were exposed to the question screen and began reading, which falls outside the scope of our primary analysis.

### 3.3. Participants

To operationalize architectural expertise, we recruited recent graduates and advanced students of architecture. All participants in the expert group had completed or were in the final semester of their degree at the same institution, providing a high degree of homogeneity in design-centered training and curricular exposure. This homogeneity improves internal validity and reduces within-group variability. From a formal perspective, these individuals qualify as architecture graduates and possess the relevant disciplinary knowledge to evaluate architectural content. Moreover, focusing on recent graduates avoids the heterogeneity seen in long-practicing professionals, whose work may diverge substantially depending on specialization (e.g., construction supervision, project management, or regulatory roles) and who may no longer engage with design-oriented tasks. Recruiting senior professionals would also present logistical difficulties, as their availability for time-intensive lab-based eye-tracking studies is typically limited. By targeting early-career experts who share a streamlined and design-focused educational experience, we maximize both relevance to the research question and consistency within the expert group.

Recruitment was conducted via university mailing lists, course announcements, and departmental networks. Participation was voluntary and compensated with either course credit (control group) or a small financial incentive (expert group). All participants provided written informed consent in accordance with the university’s ethics guidelines.

The final sample consisted of 96 participants: 48 in the expert group (trained architects or final-year Bachelor’s architecture students) and 48 in the control group (students and graduate students in language-related fields with no formal spatial training). Control participants were screened via a post-experiment questionnaire to exclude individuals with experience in architecture, visual arts, 3D modeling, gaming, or other activities involving advanced spatial reasoning.

A priori power analysis using G*Power 3.1.9.7 [33], based on effect sizes from a comparable eye-tracking study [6], indicated a required sample size of at least 44 per group (α = 0.05, power = 0.80, effect size *f* = 0.25). We increased this to 48 per group to allow for possible exclusions.

Initially, 112 participants were recruited. One architect was excluded due to a low tracking ratio (<90%), and three due to poor calibration accuracy (>0.6°). These participants were replaced. After completing the expert group recruitment, the best-matched controls were selected for final inclusion based on age and gender. Among the controls, four were excluded for low tracking ratios, seven for spatial expertise that violated inclusion criteria (e.g., gaming, artistic background, exposure to architecture through family), and five for being less optimal matches to the expert group.

The resulting groups were matched in gender (32 male, 16 female each) and age (mean = 24.4 years, SD = 1.3). All were enrolled in Bachelor’s or Master’s programs, averaging 7.1 semesters of university education. All participants were native German speakers, and the experiment was conducted entirely in German. Eye-tracking data quality averaged 97.7% (range: 92.9–99.8%), with calibration accuracy averaging 0.45° (x-axis) and 0.42° (y-axis).

### 3.4. Methods

**Figure 3:**
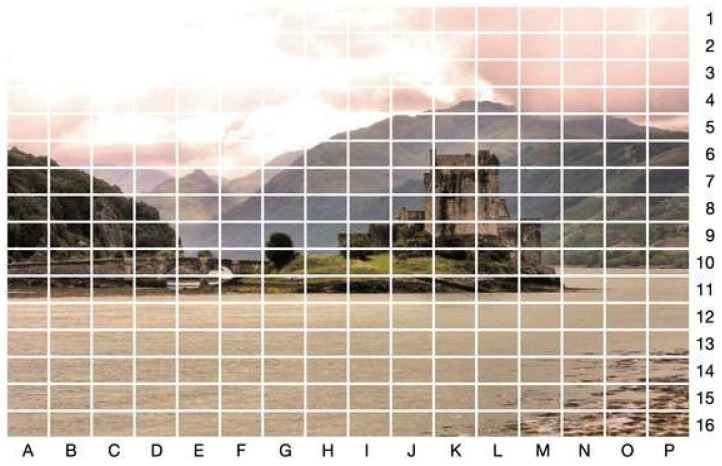
Gridded AOIs (16 × 16) overlaid on stimulus M2, resulting in 256 AOIs of equal size.

To analyze attention distribution, we used dwell times across a grid-based AOI system, a systematic approach to dividing a visual stimulus into a uniform matrix of rectangular cells. Specifically, each image was divided into a 16 × 16 grid (the highest granularity setting possible using BeGaze), resulting in 256 equal-sized rectangular AOIs per stimulus (see Figure 3). This method, known as gridded AOIs [34], enables objective quantification and content-independent analysis of visual attention distribution across the entire stimulus. The AOIs in the grid can be counted using an 8-bit variable in computing. Each AOI covered approximately 0.4% of the image (6825 pixels). Gridded AOIs provide a standardized framework for analyzing how participants engage with different regions of the stimulus without the need for predefined, feature-specific AOIs. For each stimulus and participant, we measured the total dwell time in each AOI, in comparison to the other AOIs, irrespective of the temporal order in which AOIs were visited. This method avoids biases introduced by semantic labeling or manually defined AOIs.

In theory, an image without any distinguishable features (e.g., a blank white screen) would elicit evenly distributed gaze patterns. The only exception would be the expectation of a certain center bias, acquired by experience in watching content presented on screens, where the most important content tends to be presented in the center of the screen, the action framed in the middle. In an AOI-grid with cells of equal size, participants can be assumed to spend roughly equal amounts of time on each cell of the AOI-grid. In contrast, in a picture that has one central feature, for example, a small cross in the middle of an otherwise white screen, the object would draw disproportionate attention to specific AOIs, and very little time is spent on the rest of the screen, at least until they get bored with the one central feature, decreasing distributional uniformity. Exploiting this behavior, psycholinguistics studies use a fixation cross to ensure the attention of the participants is in the middle of the screen before presenting a new stimulus. In real pictures, participants spend more time on interesting parts of the picture, and less time on the background or features that they do not find interesting. Figure 3 illustrates the AOI grid overlaid on a stimulus from the Mixed category. The grid is organized vertically into rows numbered 1 to 16 and horizontally into columns labeled A to P, similar to an enlarged chessboard.

While we could have counted the number of AOIs visited, this approach fails to differentiate between brief glances and sustained attention. Similarly, using means, medians, or modes of dwell time fails to fully capture the distribution of attention and is sensitive to outliers and non-normal distributions. In addition, means are affected by outliers, medians by non-normal distribution, and modes by heavy tails. A measure that can deliver one single number for visual exploration and at the same time overcome all these statistical shortcomings is Shannon’s entropy [35]. Entropy measures the absence of order, i.e., the presence of randomness. If participants spend equal amounts on each AOI of the grid, as in the example of the white screen, we get a homogenous distribution and thus high entropy. If, in contrast, all participants only look at one AOI cell, we have the highest possible order and no entropy. Imagine you are searching for the house keys in a messy, chaotic room versus in a tidy and organized room. In the messy room, your eyes would be darting all over the place, visually searching. This visual behavior would get a high entropy measure because there is no clear focus. But in the tidy room, you could direct your gaze right to the hook where the house keys are usually kept. This behavior would give a low entropy measure.

In technical terms, entropy measures the number of bits that are required to describe the irregularity found in the data. Shannon’s Entropy (*H*) measures the degree of absence of order, that is, how non-homogeneous a distribution is:

(1) $$H=-\sum_{i=1}^{N}p_{i}{log}_{2}\left(p_{i}\right)$$

where *N* is the number of AOIs and *p_i_* is the relative frequency of visits. If *p_i_* = 0 for some *i*, then *p_i_ log*_2_(*p_i_*) = 0 is set as a convention. An easily interpretable measure is relative entropy, defined as follows if there is random distribution:

(2) $$H_{rel}=\frac{H}{H_{max}},where{\;H}_{max}={log}_{2}\left(N\right)$$

Relative entropy equals 1 when attention is evenly distributed across AOIs, and approaches 0 when gaze is concentrated in a few AOIs. Zipfian distributions typically yield values around 0.7 [36]. Relative entropy depends on the randomness of the distribution of types (if each type has equal token frequency, entropy is highest), but also the number of types (more types lead to higher entropy). Our AOI grid always has the same number of types (8 × 8 = 256). We will mainly report absolute Shannon entropy (*H*), but relative entropies can always be calculated. Unlike related but simpler methods such as type/token ratio, Shannon’s entropy is not very sensitive to outliers, because their probability mass is small, thus their contribution to the entropy remains limited.

Entropy is at the cross-road between descriptive statistics and model statistics. It can be used as a descriptive measure, but it is also an indicator of model fit [37], and often provides the loss function that is minimized during model fitting [38,39]. In our case, we assess the fit to a model of fixation on mainly one AOI. We also report results using inferential statistics and assess whether differences are strong enough for prediction models using logistic regression.

A popular visual representation of order versus entropy is heat maps. While individual heat maps are easy to interpret, a systematic comparison of how much heat (i.e., order) an individual picture shows in comparison to another picture, or whether one group of participants (architects) shows more or less variance in heat than another group (laypeople), is tedious if not confusing.

A low entropy indicates a trend for participants to focus particularly strongly on a few selected features in the picture, probably because they find it particularly interesting; high entropy indicates that the participants explore all aspects of the picture (systematically or not). We decided to analyze a relatively short time segment (seconds 0.25 to 6) because bored participants could also either absent-mindedly stare at a random part of the picture (which leads to low entropy) or restlessly wander across the picture (which leads to high entropy). The entropy is calculated for each stimulus and each participant, over the times between 0.25 and 6.00 s.

#### 3.4.1. Visualization with Histograms and Densities

Summarizations abstract from the individual data, for example, though heatmaps of pictures to more easily comparable measures. Shannon’s Entropy offers a single number, but it leaves the distribution of the time spent underspecified. In order to interpret differences in entropy, we use histograms and density plots to reveal the peaks—i.e., the mode—and the tails and maxima in the distribution of participant-level entropy values. A low mode indicates that many participants skip many AOIs in the grid, while a long right tail, or even a high maximum, reveals that some participants find one AOI cell particularly interesting. For clarification, we will first show a histogram to visualize the discrete intervals. Later, we will mainly use densities, displayed using idealized continuous curves.

#### 3.4.2. Skewness

We have no fixed expectation of the distribution of the times spent per AOI shown by these histograms and density plots. A homogenous distribution (highest entropy) would be characterized by a normal distribution, while high order (low entropy) would lead to a Zipfian distribution with most AOIs showing a dwell time of 0 or a very low number.

As we do not expect normal distribution, measures used to describe how far the distributions deviate from normality can also reveal patterns. Skewness is one of such measures. Skewness describes how much, in comparison to a perfect normal distribution, our data is left- or right-distributed. As order in our data leads away from a normal to an increasingly Zipfian distribution, we expect a strong right distribution, i.e., a left peak and strong outliers far on the right, particularly in the pictures in which certain features kindle a strong interest.

Skewness is defined as

(3) $$skewness=\frac{\sum_{i=1}^{N}{\left(Y_{i}-\bar{Y}\right)}^{3}/N}{s^{3}}$$

where *Ȳ* is the mean, *N* the number of observations and *s* is the standard deviation. A value of 0 indicates normal distribution, left distributions are negative, and right distributions positive.

#### 3.4.3. Kurtosis

While skewness allows us to assess a left or right distribution, kurtosis measures the heaviness of the tail. A heavy tail would arrive in a distribution containing many high, and some extremely high values, due to some visual features that participants focus on particularly strongly. Kurtosis is defined as

(4) $$kurtosis=\frac{\sum_{i=1}^{N}{\left(Y_{i}-\bar{Y}\right)}^{4}/N}{s^{4}}-3$$

Positive values express “excessive” heavy tails. We can use this measure as a proxy for how interesting participants find certain features in the picture.

## 4. Results

This section presents the results, organized by overall trends, patterns across the three scene categories (built, mixed, and natural environments), and selected individual stimuli. We begin with aggregate trends in visual attention among architects and laypeople and then illustrate these with representative examples. Eye-tracking data were analyzed for the first 6 s of free viewing, excluding the first 250 ms, during which attention was artificially centered by a fixation cross. Thus, 0 on the timeline actually corresponds to the 2500 ms mark.

### 4.1. Overall Trends

**Figure 4:**
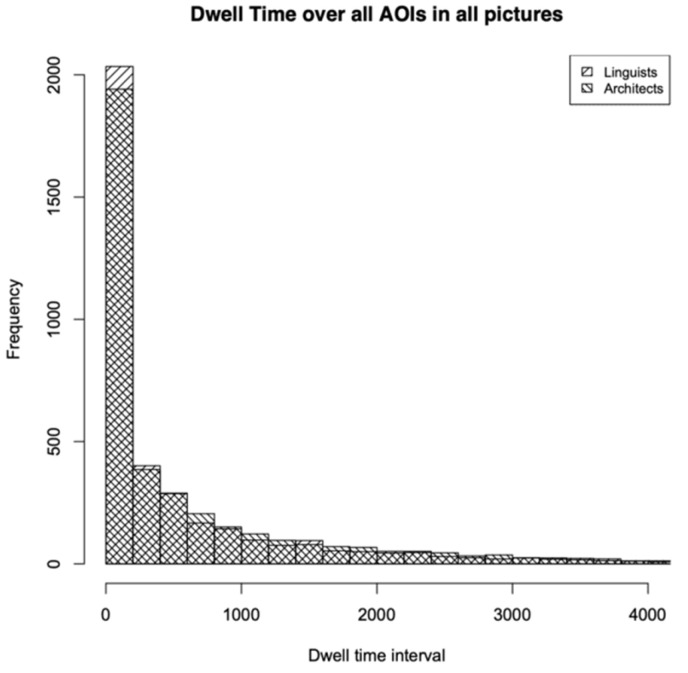
Overall trend. Comparison between groups for all participants and all stimuli.

To quantify the general distribution of visual attention, we first aggregated dwell times for each AOI across all 48 participants and all 15 stimuli. Figure 4 shows a histogram comparing this general aggregation for both groups. The discrete intervals are shown in the histogram. In following visualisations, only densities that display idealized continuous curves will be represented. Figure 4 shows that the distribution is closer to a Zipfian value than to normal for both groups, which means that, in all pictures, certain areas attracted more attention than others. Laypeople showed more AOI regions with no or minimal dwell time, whereas architects exhibited a broader distribution of attention, with more regions receiving moderate to high dwell times. Notably, the range from 800 ms to 2000 ms featured substantially more entries for architects. This is also in line with the fact that 43% of the laypeople’s AOIs have a dwell time of 0 ms, opposed to 41% in the case of the architects. Since laypeople have a more dominant class of dwell time = 0 ms, their entropy is also a bit higher. Table 1 summarizes key values.

The more dominant value of dwell time = 0 leads to a lower median for the laypeople, while (unexpectedly) the mean is slightly higher, due to several outliers. Skewness is higher for the architects, due to a more Zipfian distribution, which indicates that certain features draw their visual attention. Kurtosis is much higher for architects, due to heavier tails. The value of the Pearson correlation is challenging to interpret in this context, as we are comparing multiple stimuli that vary significantly from one another. Because the stimuli vary widely in content and complexity, the Pearson correlation across all AOIs should be interpreted cautiously and is included here primarily for completeness.

### 4.2. Individual Stimuli

**Figure 5:**
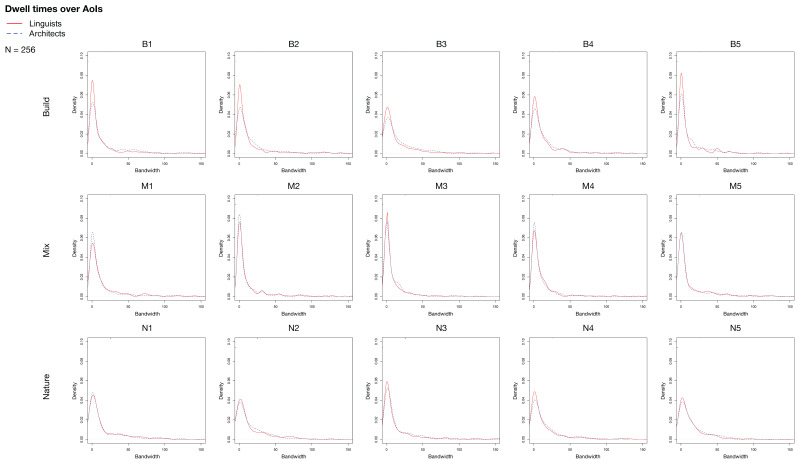
Dwell time densities for each of the 15 stimuli arranged as small multiples.

After providing a broad overview of the eye tracking data aggregated across all 15 stimuli, we now take a closer look at each individual stimulus. The stimuli are grouped into the categories “built environment”, “natural environment”, and “mixed environment”, depending on the content of the presented images. We were able to identify general trends for each category. The density curves for each individual stimulus are displayed in Figure 5.

**Figure 6:**
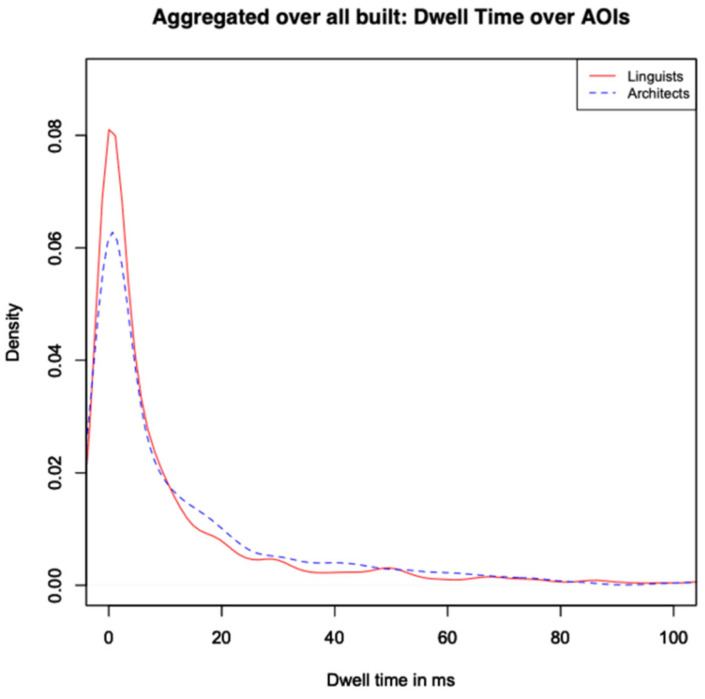
Distribution of dwell time for all built environment stimuli.

Across all built environment stimuli, similar trends emerge: Entropy is higher and standard deviation is lower for architects, indicating that they leave fewer parts of the picture unexplored. The means are similar, and the median is higher in the case of architects. Architects’ distributions show reduced skewness and kurtosis, reflecting less sharply peaked dwell-time distributions and more delayed attention allocation across AOIs. Architects also have a lower standard deviation than laypeople. Since out of the three categories, built environments are the most relevant for architecture, we visualize the distribution of the density for all five built environment stimuli, aggregated in Figure 6, and present the measures summed over all built environment stimuli in Table 2. In contrast, the mixed environment stimuli elicited more focused visual attention from architects, as indicated by pronounced early peaks and reduced entropy, indicating that they concentrate on a smaller number of AOIs (the ones with salient architectural elements). Skewness and kurtosis are about the same for both groups. Still, entropy is slightly lower for architects. For the natural environment stimuli, architects show slightly more pronounced peaks around zero than the control group, with the exception of N1. This is difficult to explain, since N1 and N2 are quite comparable. It is noteworthy that, in this type of stimulus, the entropy is the lowest for both groups, and the differences between the two groups are the smallest.

Comparing across the natural environment stimuli does not lead to clear trends. The natural environment category was included as control to see how architects visually scan a scene when no architectural elements are to be seen.

### 4.3. Selected Individual Stimuli

To show how participants look at individual stimuli, one picture from each category was selected as an example: B1, M2 and N2. The summary statistics for the three chosen examples can be found in Table 3. Entropy was consistently higher for architects across all three categories, suggesting more distributed visual exploration and more complex scanpaths as compared to laypeople. Architects also display slightly higher relative entropy values compared to laypeople, indicating a greater focus on specific features. The mean fixation times are similar between architects and laypeople, with minor variations. Laypeople generally show higher variability in fixation times compared to architects. This may indicate more inconsistent viewing patterns among laypeople. Architects tend to have slightly higher median fixation durations than laypeople (except in the case of stimuli M2 Scotland, where the median for architects is 0.0 ms compared to 0.7 ms for laypeople). This may indicate different scanning strategies or priorities. Laypeople exhibit higher skewness in fixation durations, indicating a more uneven distribution, with occasional long fixations. Laypeople also show significantly higher kurtosis, reflecting a distribution with more extreme outliers. In contrast, architects demonstrate more uniform fixation behavior. In summary, architects generally demonstrate more systematic and consistent visual behavior, reflected in higher entropy and lower variability. Laypeople, on the other hand, show more dispersed and irregular viewing patterns, as seen in their higher standard deviations, skewness, and kurtosis values. These differences likely reflect the distinct cognitive strategies employed by architects and laypeople when analyzing visual scenes.

#### 4.3.1. A Picture from the Built Environment Category: B1 (St. Louis)

**Figure 7:**
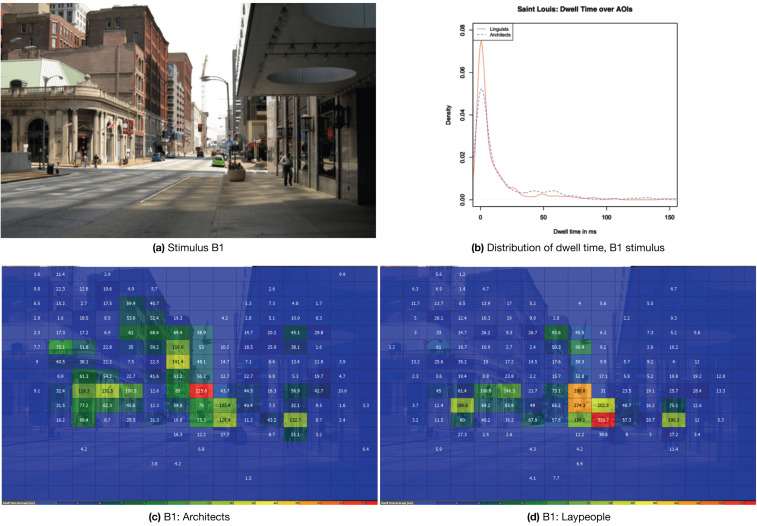
(**a**) Stimulus B1 “St. Louis”; (**b**) the distribution of dwell times; (**c**,**d**) heat maps for both groups while viewing the stimulus.

The chosen example for the built environment category is stimulus B1, a picture showing a street in the American city of Saint Louis. The Stimulus is reproduced in Figure 7a,c,d shows heatmaps for both the architects and the laypeople separately, and Figure 7b shows the visualization of the density for this stimulus. The summary statistics for B1 can be found in Table 3. The heat maps indicate that laypeople disproportionately focused on the parked green car, which is an uncommon color for vehicles. Regarding the density, the group differences in B1 are more pronounced than the average differences across the full stimulus set, which also results in a lower Pearson correlation. In particular, the peak around zero is more pronounced for laypeople. This pronounced peak contributes to the high skewness observed in the laypeople’s data. The high kurtosis is also anticipated by the fact that the mean is higher, although the median is lower, and also the maximum and the standard deviation for the laypeople are higher.

#### 4.3.2. A Picture from the Mixed Environment Category: M2 (Scotland)

**Figure 8:**
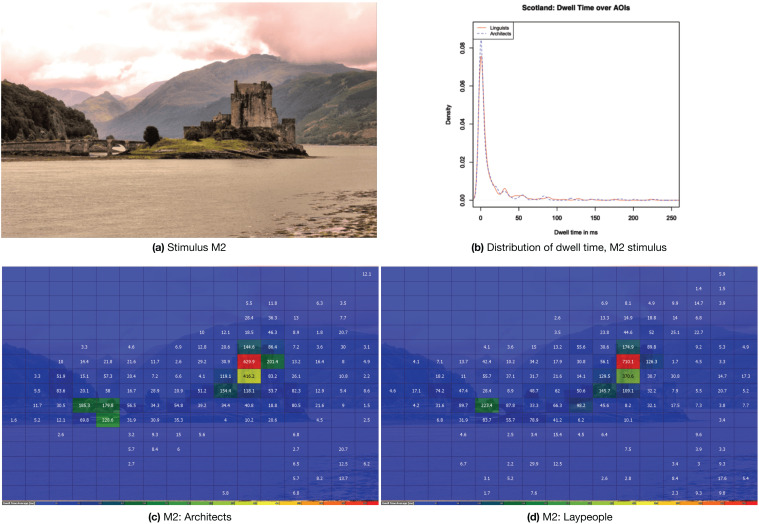
(**a**) Stimulus M2 “Scotland”; (**b**) the distribution of dwell times; (**c**,**d**) heat maps for both groups while viewing the stimulus.

The chosen example for the mixed environment category is stimulus M2, a picture showing a castle in the highlands of Scotland. The stimulus is reproduced in Figure 8a Figure 8c,d shows heatmaps for both the architects and the laypeople separately. The heat maps indicate that while both groups are primarily interested in the castle, the architects tend to focus on the bridge leading to the castle. This bridge represents an additional architectural element that complements the primary subject of the image. Figure 8b shows the visualization of the density for stimulus M2. The summary statistics for M2 can also be found in Table 3. For stimulus M2, entropy is generally lower than in both other exemplary scenes. The architectural feature attracts much attention, so that very many, in the case of the architects, even the majority of AOIs are not visited (median = 0). The Pearson correlation is very high, as both groups have a trend to skip many AOIs and only concentrate on a few selected ones. The maximum dwell times (710 ms for laypeople and 629 for architects) are more than twice as high as in the two other exemplary pictures. The arising distribution is much further away from a normal distribution than in the previous pictures. The peak is higher, but there is still considerable mass very far to the right, which is why up to 250 ms are displayed in this graph. Despite the similarities between experts and laypeople, we can also notice some differences: entropy is slightly lower for architects, unlike in the other two exemplary pictures, and the architects are drawn even more strongly towards the built areas.

#### 4.3.3. A Picture from the Natural Environment Category: N2 (Canada)

**Figure 9:**
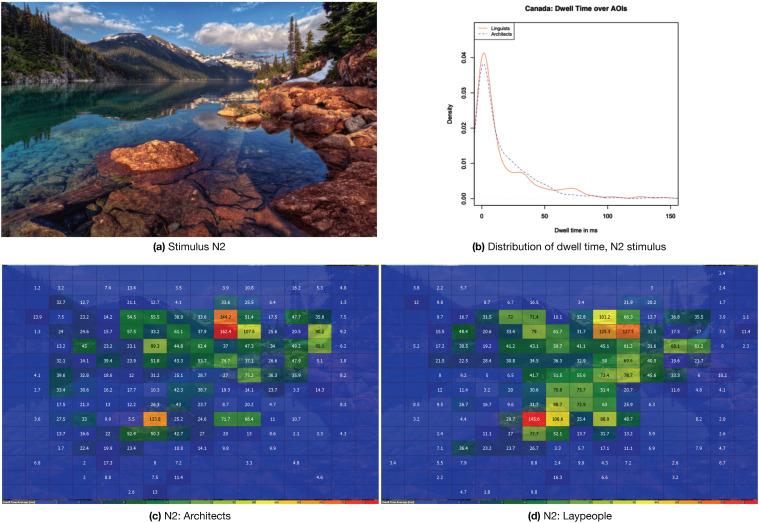
(**a**) Stimulus N2 “Canada”; (**b**) the distribution of dwell times; (**c**,**d**) heat maps for each group while viewing the stimulus.

The chosen example for the natural environment category is stimulus N2, a picture showing a lake in Canada. The stimulus is reproduced in Figure 9a,c,d, showing heatmaps for both the architects and the laypeople while looking at this stimulus. The heat maps indicate that, while both groups are primarily interested in the rock in the foreground and the vanishing point, the architects focus more on the vanishing point and the laypeople on the foreground. Figure 9b shows the visualization of the density for stimulus N2. The summary statistics for N2 can also be found in Table 3 below. Entropy is generally higher than in the B1 picture, skewness and kurtosis are smaller, which indicates a smaller focus on a few interesting AOIs. The much lower peak and the higher medians also show that fewer parts are missed, and fewer parts are of particular interest. While the differences to the other categories are marked, the differences between laypeople and architects are small. This results in a higher Pearson correlation between the two groups.

### 4.4. Significance Testing

Not all of the observed differences are significant. Often, there are not enough participants to reach significance for an individual picture. Limited generalizations are possible, though. We categorized AOIs as either visually salient (‘focus areas’) or less informative (‘white space’). Laypeople spent significantly more time viewing white space (paired *t*-test, *p* < 0.5%). At the aggregated level, when summing all participants of each group, differences are highly significant. If total dwell time between all AOIs between architects and laypeople is compared for a given picture, the differences are highly significant according to the chi-square contingency test. (To meet the requirements of the chi-square test, we had to filter the dwell time totals of AOIs in which both groups had zero values.)

### 4.5. Prediction by Model

The eye tracking data and the models learned from them can inform predictive models distinguishing between architects and laypeople. At the aggregate level of the entire group, the differences are strong enough to distinguish between architects and laypeople in all cases. The differences are highly significant (*p* < 2.2 × 10^−16^), according to the chi-square contingency test, comparing the dwell time total between the laypeople and architects.

In contrast, at the level of the individual participant, prediction is hard. We have tested predictability with a regression model (logistic regression). Model accuracy is lower. For the example of B1 (St. Louis) we obtain 68% prediction accuracy. While this beats the random baseline, it would be too low for practical applicability. Our main aim is not to predict if a given viewer is an architect or a layperson, but to characterize and describe typical differences between groups. Given more data, it can be assumed that prediction of individuals would improve. Even with a high-performing classifier, the identification of specific predictive AOIs offers limited interpretability. In contrast, entropy provides a more intuitive and theoretically grounded measure of visual behavior.

## 5. Discussion

Despite increasing interest in the cognitive underpinnings of architectural perception, empirical research in this domain often suffers from low ecological validity and ambiguous interpretability. A particularly underexplored area concerns the mechanisms that guide visual attention in experts versus laypeople when viewing architectural scenes. By comparing entropy metrics between architects and non-experts, our study uncovers systematic differences in visual search behavior and provides a quantifiable basis for understanding how professional training shapes perceptual strategies. In the current study, we investigate the visual exploratory behavior of architects compared to a laypeople group, by measuring the two groups’ eye movements while looking at landscape photographs with varying degrees of architectural elements. We analyze the distribution of their visual attention using gridded AOIs.

### 5.1. Summary of Main Findings

The data reveal that architects allocate visual attention differently from laypeople. They focus more intensively on built structures and exhibit lower entropy in their gaze distributions, indicating less random scanning and more systematic exploration. Their gaze patterns are also more homogeneous across individuals, suggesting shared perceptual strategies shaped by training. In mixed scenes (e.g., landscapes with individual buildings), both groups looked at similar regions, but architects concentrated more on architectural elements. These differences were not observed in natural environments, highlighting the domain-specific nature of architectural expertise.

Interestingly, while architects displayed consistent differences in visual attention when viewing images set in urban landscapes, these patterns were absent in natural landscapes, suggesting their perceptual distinctions are domain-specific. As expected, in photographs not containing any architectural elements, such as the pictures in the natural environment category, no differences regarding the utilization of grammar of space were found between the two groups. These findings align with prior eye-tracking studies by Mertins et al. [6], who showed that architects preferentially attend to façades and structural cues, while laypeople focus on human figures and objects at eye level. However, our study extends this line of research by systematically manipulating the degree of architectural content across three categories of stimuli (built, natural and mixed). In contrast to Kosel et al. [10], who interpreted higher entropy as an indicator of more complex cognitive strategies in teachers, we show that entropy must be interpreted in context. For architects, low entropy in mixed scenes likely reflects goal-directed focus, not cognitive simplicity. In this way, our results provide further evidence that visuospatial perception of architectural space is affected by expertise, but our results suggest that the interpretation of entropy depends on context. In built environments, architects show higher entropy but lower variability, suggesting broad yet systematic exploration of architectural features. In mixed environments, however, lower entropy reflects a focused interest in specific architectural elements. These contrasting patterns highlight the dual nature of architectural expertise: broad scanning when needed, and concentrated attention when salient cues are present.

### 5.2. Interpretation of Entropy Measures

Among the metrics tested, Shannon’s entropy, its standard deviation, and kurtosis emerged as the most informative for differentiating expert and layperson gaze behavior. Mean fixation time, in contrast, remained stable across conditions and groups. While entropy provides a powerful lens for quantifying visual exploration, it must be interpreted contextually and in conjunction with spatial patterns and stimulus content. As our results show, similar entropy values can result from qualitatively different strategies, underscoring the need for multi-dimensional analysis. The interpretation of the entropy variables is not straightforward, which underscores the value of the insights provided in this project. However, the interpretation we propose diverges from that of Kosel et al. [10], who use Shannon’s entropy to analyze the eye movements of teachers. Their findings indicated that teachers exhibit higher entropy coefficients, which they associated with more complex scan-paths.

Rather than just claiming that expertise influences visual attention, the objective of this paper is to examine whether these differences can be measured and characterized, especially because the differences themselves are not particularly large. We explored various variables related to entropy to determine which variable best reflects the particular way in which architects observe architectural content as compared to lay people. Our conclusion is that entropy is a useful and effective measurement of differences in the temporal allocation of visual attention between experts and laypeople. We did not intend to build a classifier that can distinguish between architects and laypeople, but to describe, characterize, and interpret the typical differences. Given enough data, a classifier would also lead to better results.

Shannon’s concept, in essence, posits that in contexts where probabilities are highly unequal, entropy is low, reflecting reduced uncertainty. Conversely, when many outcomes have approximately equal probabilities, entropy is high, indicating maximum uncertainty. The interpretation we propose is that low entropy suggests a tendency for viewers to focus intensely on a few selected features in an image, likely because they find these features particularly relevant. In contrast, high entropy indicates that participants explore the image more broadly, attending to multiple aspects. A low mode suggests that many participants skip numerous areas of interest within the grid, while a long right tail or a high maximum value indicates that certain participants find a few AOIs especially interesting.

### 5.3. Grammar of Space and Expertise

Drawing on theories like “Seeing-for-saying” [29,30] and “Seeing-for-Speaking” [25,26,27,28], which posit that language expertise influences cognitive processes relevant to linguistic tasks, we extend this principle to non-linguistic domains. While these theories focus on the interplay between language and cognition, we consider language as one of many cognitive skills humans can acquire and master. Given humans’ extensive exposure to language, it is arguably one of the most developed areas of expertise. However, as individuals acquire additional expertise throughout their lives, other domains, such as architecture, may similarly shape cognitive processes in ways that are specific to their respective contexts. We extend the idea of structured perceptual schemata to the visual domain of architecture. Just as language learners internalize grammatical rules for producing coherent speech, architects may acquire a “grammar of space”: a system of perceptual rules that guide attention toward functionally and aesthetically salient elements in a scene. This analogy frames architectural perception as a learned, structured process, not merely a product of innate visual salience.

In this study, we designed a framework that varies architectural expertise while holding other expertise domains, such as language, constant. Our primary research question examines whether architectural expertise influences unconscious visual processing of architectural imagery and if the experts’ visual architectural processing can be measured. We hypothesize that years of architectural training led architects to internalize specific patterns of visual attention, adhering to a “grammar of space” that governs their perception of architectural content, even when performing tasks not overtly architectural in nature.

The way human beings experience space and the way it is encoded grammatically in language are not necessarily universal, but influenced not only by human physical configuration and neurophysiological apparatus, but also by cultural experiences. This complex interplay between universal biological and cognitive constraints and cultural-linguistic variation in spatial representation is generally referred to as the grammar of space [40]. Languages vary a lot cross-linguistically with regard to which spatial relations they encode and with which grammatical means they do so (for example, using prepositions, specialized verbs, etc.). So, although the cognitive foundations of humans and the physicality of perceiving and interacting with space are fairly universal, the cultural and linguistic experiences of different groups of people can greatly influence how they experience and interact with their environment. For instance, living in mountainous vs. flat terrain may influence the spatial grammar of the languages spoken in these areas. In the case of the Northern Alta language [41], spoken in a mountainous region of the Philippines, the morphological system of the language has different spatial subdomains (location, disposition, and orientation) encoded through specific derivational affixes and lexical roots with spatial meanings such as front, side, top, and back to derive a variety of spatial verbs that are key for expressing space relations in that language. The grammatical differences between languages can compound, resulting in different information being selected for expression and similar spatial information being linguistically packaged in very different ways, even in everyday linguistic tasks such as giving route directions [42].

### 5.4. Limitations and Future Work

A primary limitation is the sample’s restricted generalizability: most architect participants were recent graduates from a single institution, potentially limiting the range of expertise. In addition, the exclusive use of 2D photographs fails to fully capture the immersive, 3D nature of architectural experience. While architects frequently work with two-dimensional representations, such as blueprints and CAD software, their professional practice ultimately engages with three-dimensional space. Future work should incorporate 3D, VR or AR environments to approximate real-world spatial exploration and expertise in a three-dimensional context. This approach would significantly improve the relevance of findings and would allow for the examination of how experts interact with architectural spaces in a more immersive manner. For instance, participants could engage with virtual or physical models of buildings rather than static photographs, enabling a deeper understanding of perception in spatially complex environments. Similar to the approach of Benz and Rambow [5], who asked architects and laypeople to provide oral evaluations of exposed concrete use in situ (instead of using pictures of the buildings), incorporating VR could offer valuable insights into the multi-sensory nature of architectural expertise while keeping the procedure controlled and comparable within subjects. These findings also open avenues for investigating how expertise in other disciplines influences perception.

A methodological limitation of the present study concerns the definition of the AOI grid. While the 16×16 grid-based AOI system enabled an objective, content-independent analysis of gaze distribution by dividing each image into 256 equal-sized cells—the highest granularity available in the BeGaze software—this fixed grid size may inherently constrain the precision with which highly fine-grained visual behaviors are captured. This limitation is particularly relevant for expert attention to subtle architectural details. Future research could investigate the effects of varying grid granularities or employ post hoc AOI definitions (derived from participants’ fixation patterns) to better capture nuanced attentional shifts. Such approaches would facilitate a more detailed characterization of the “Grammar of Space.”

Another constraint lies in our analytic scope: while we focused on entropy and related distributional measures, future studies could benefit from predictive modeling to develop classifiers that distinguish expert from non-expert gaze behavior. We believe that the results that we obtained in characterizing the features are valuable, nevertheless. Instead of focusing on which AOIs are particularly interesting for architects, they showed us that their gaze patterns are different.

## 6. Conclusions and Broader Implications

We propose that the prolonged exposure and targeted education of architects contribute to architects acquiring a grammar of space. This conceptual framework, as previously described [6,7,40], directs experts’ visual attention towards specific spatial elements and structures, such as facades and volumetric features of buildings. Furthermore, this grammar shifts architects’ perspective beyond ground level, encouraging them to focus on spatial elements not typically of interest to laypeople.

Our findings support the view that domain-specific expertise fundamentally shapes visual perception. Architects, shaped by years of training, exhibit a distinctive internalized “grammar of space” that directs their attention to volumetric, structural, and contextual architectural features. This internalized perceptual system enables them to process spatial scenes efficiently and selectively, even when tasks are not explicitly architectural.

Unlike non-experts, who might focus on decorative elements, architects subconsciously prioritize the functional and spatial aspects that define a building’s role within its environment. This dual capacity to absorb broader scenes while honing in on specific details reflects a heightened visual acuity related to their expertise. This research underscores the adaptability of the human brain, where prolonged exposure and training in a specific domain foster specialized perceptual mechanisms. For architects, this results in a subconscious, efficient processing of spatial information, akin to fluency in a native language. The same way that your native language shapes the way you think, their expertise shapes the way architects perceive architectural elements, even when the task at hand is not overtly related to architecture at all. By understanding the cognitive processes underlying architects’ perception, we gain a greater appreciation for the depth of expertise involved in designing spaces that are not only functional and aesthetically pleasing but also harmoniously integrated in their environments.

Entropy-based metrics offer a robust, assumption-free method to quantify these differences, paving the way for future research into how expertise across domains modifies perceptual mechanisms. Crucially, this method avoids assumptions about where viewers are expected to look, making it especially suitable for contrasting experts and non-experts whose perceptual strategies may diverge. By using entropy, we aim to capture the fluid and individualized nature of visual exploration in complex visual scenes.

More broadly, this research contributes to ongoing efforts to objectify design evaluation of architectural quality (both aesthetic and functional) by linking perceptual behavior to training and cognitive style. In this way, this line of research not only advances theoretical models of visual cognition but also holds practical relevance for the design and evaluation of architectural spaces that are responsive to the perceptual priorities of diverse user groups.

## Figures and Tables

**Table 1:** Measures added across all stimuli.

Measure	Architects	Laypeople
Entropy	7.016	6.923
Relative Entropy	87.71%	86.54%
Mean	816.6 ms	817.0 ms
Standard Deviation	2100 ms	2161 ms
Median	192.0 ms	163.9 ms
Skewness	13.73	11.40
Kurtosis	358.1	246.4
Pearson Correlation	0.896	

**Table 2:** Summary statistics for all stimuli from the category “built environment”.

Measure	Architects	Laypeople
Shannon’s Entropy	5.796	5.581
Relative Entropy	57.97%	55.18%
Mean	17.08 ms	17.15 ms
Standard Deviation	34.38 ms	44.34 ms
Median	4.1 ms	3.2 ms
Skewness	4.732	5.961
Kurtosis	33.68	48.35
Pearson Correlation	0.830	

**Table 3:** Summary statistics for the exemplary stimuli B1, M2 and N1 in comparison.

	B1: St. Louis		M2: Scotland		N1: Canada	
Measure	Architects	Laypeople	Architects	Laypeople	Architects	Laypeople
Shannon’s Entropy	4.420	4.090	3.831	3.901	4.667	4.595
Relative Entropy	55.25%	51.11%	47.89%	48.76%	58.34%	57.44%
Mean	17.00 ms	17.11 ms	17.72 ms	17.56 ms	16.40 ms	16.76 ms
Standard Deviation	30.54 ms	42.62 ms	56.39 ms	56.80 ms	24.51 ms	25.83 ms
Median	2.65 ms	2.2 ms	0.0 ms	0.7 ms	6.6 ms	4.75 ms
Skewness	2.88	4.47	7.08	8.45	2.62	2.13
Kurtosis	10.99	23.20	63.15	90.34	9.35	4.91
Pearson Correlation	0.770		0.945		0.819	

## Data Availability

The original data presented in the study are openly available at https://osf.io/6xbgp/ at DOI 10.17605/OSF.IO/6XBGP.

## Supplementary Materials

The following supporting information can be downloaded at Stimuli, Results and RAW data: https://osf.io/6xbgp/ (accessed on 26 May 2025).

## Abbreviations

The following abbreviations are used in this manuscript:

AOI	Area of Interest
CAD	Computer-aided Design
VR	Virtual Reality
AR	Augmented Reality
MRI	Magnetic Resonance Imaging
LCD	Liquid-crystal display
